# Simultaneous Rapid Determination of Seven *Alternaria* Toxins in Tuberous Crops during Storage Using QuEChERS Coupled with Ultrahigh-Performance Liquid Chromatography-Tandem Mass Spectrometry

**DOI:** 10.3390/foods12040862

**Published:** 2023-02-17

**Authors:** Jiali Xing, Xi Wu, Xiaorong Xu, Hai Cheng, Jian Shen, Ruihang Zheng, Lingyan Mao, Xiaohu Luo, Yinghua Mu, Yu Liu

**Affiliations:** 1 Ningbo Academy of Product and Food Quality Inspection (Ningbo Fibre Inspection Institute), Ningbo 315048, China; 2 College of Food and Pharmaceutical Science, Ningbo University, Ningbo 315000, China

**Keywords:** UPLC–MS/MS, multiplex determination, *Alternaria* toxins, tuberous crops

## Abstract

Robust and sensitive ultrahigh-performance liquid chromatography–tandem mass spectrometry (UPLC–MS/MS) combined with the quick, easy, cheap, effective, rugged, and safe (QuEChERS) method was applied for the detection of seven *Alternaria* toxins (*A*Ts) in tuberous crops. The influence of tuber conditions (fresh, germinated, and moldy) during storage on the concentration of the seven *A*Ts is also investigated. *A*Ts were extracted with acetonitrile under acidic conditions and purified with a C_18_ adsorbent. *A*Ts were scanned with electrospray ionization (positive/negative ion) dynamic switching and detected in MRM mode. Calibration curve analysis results reveal good linear relationships in all toxin concentration ranges (*R*^2^ > 0.99). The limit of detection and limit of quantification were 0.25–0.70 and 0.83–2.31 μg/kg, respectively. The average recoveries of the seven *A*Ts were 83.2–104% with intra-/inter-day precision at 3.52–6.55% and 4.02–7.26%, respectively. The developed method provided adequate selectivity, sensitivity, and precision in detecting the seven *A*Ts at trace levels, and dispensed with standard addition or matrix-matched calibration to compensate for matrix effects. *A*Ts in the fresh, germinated, and moldy samples of tuberous crops in storage (taro, potato, sweet potato, yam, cassava) were analyzed with this method, and the concentrations were 2.01–14.51 μg/kg and significantly increased with storage duration. ALS was detected in most samples, whereas no quantities of ALT and ATX-I were detected. AME was often detected in combination with AOH in sweet potatoes. TeA and Ten were mostly detected in taro, potato, and yam. The established method could be used for the simultaneous detection and quantification of multicomponent toxins in elaborate matrices.

## 1. Introduction

Tuberous crops are an important global crop category, only second to wheat, rice, and corn. Tuberous crops are a staple food in many countries [1]. There are different kinds of tuberous crops, including taro, potato, sweet potato, yam, and cassava, which are used in traditional staple foods (e.g., steamed bread, noodles, rice noodles) and cereal-based food. Tuberous crops are highly susceptible to *Alternaria* fungi under various climatic conditions [2,3]. *Alternaria* fungi infect tubers, causing black or brown spots on the tuber surface that are difficult to remove with washing and sorting, eventually evolving into tuber rot that significantly affects crop yield and quality [4]. Being an omnipresent fungal genus, *Alternaria* spp. can cause pre- and postharvest damage to agricultural products, especially high-water-content and nutrient-rich cereal grains, fruits, and vegetables [5,6]. *Alternaria* toxins (*A*Ts) are fungal metabolites generated by *Alternaria* in polluted food that can severely affect animal and human health. The European Food Safety Authority (EFSA) uses the threshold of toxicological concern (TTC) approach to assess the human health effects of some *A*Ts. For genotoxic AOH and AME, 95% of the estimated mean dietary exposures exceeded the TTC value (2.5 ng/kg body weight (bw) per day), while for nongenotoxic TeA and Ten, the TTC value was 1500 ng/kg bw/day [7]. *A*Ts can seriously threaten human health when consumed in excess of the TTC value, resulting in salivation, vomiting, erythema, convulsions, gastrointestinal hemorrhage, and immunosuppression [8,9]. Therefore, the accurate and quantitative detection of *A*Ts in food including tuberous crops is increasingly important not only for research projects, but also risk evaluation studies [10,11].

**Figure 1:**
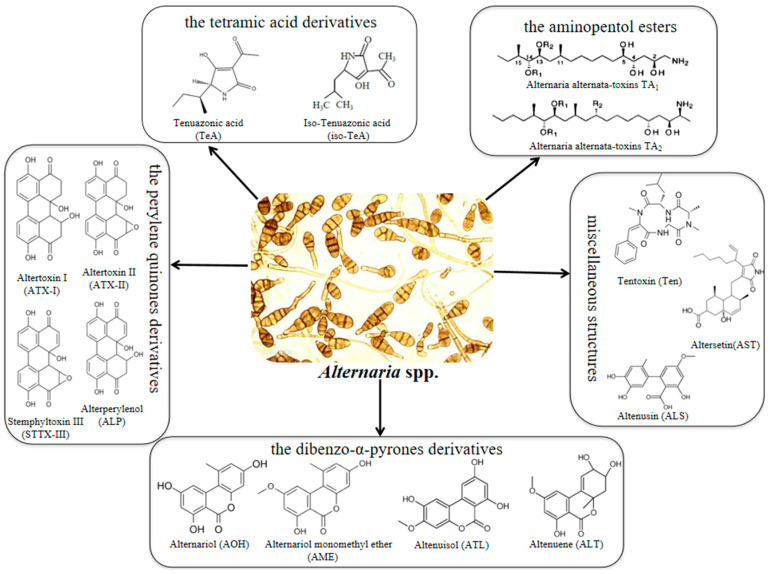
Chemical structure of *Alternaria* toxins.

These contaminants principally pertain to five different chemical categories (Figure 1) [7,12]: (1) dibenzo-α-pyrones derivatives, including alternariol (AOH), alternariol monomethyl ether (AME), and altenuene (ALT); (2) tetramic acid derivatives, e.g., tenuazonic acid (TeA) and iso-tenuazonic acid (iso-TeA); (3) perylene quinones derivatives, the main members of which are altertoxin (i.e, ATX-I, ATX-II and ATX-III), stemphyltoxin (STTX-III), and alterperylenol (ALP), which yield large amounts at the preharvest section [13,14]; (4) aminopentol esters, such as *A. alternata* f. sp. *lycopersici* toxins (AAL toxins); (5) disordered structures on behalf of tentoxin (Ten), altenusin (ALS), and altersetin (AST).

The most prevalent *A*Ts are mainly focused on AOH, AME, TeA, and Ten, whereas the occurrence of other *A*Ts is fairly scarce, mostly owing to the deficiency of analytical methodologies [15]. Furthermore, the detection of *A*Ts has focused on cereals and fruits [16]. For instance, Gotthardt et al. [16] developed a systematic analytical technology for eight *A*Ts (AOH, AME, TeA, Ten, ATX-I, ATX-II, ALTP, and STTX-III) in single-/multigrain goods (spelt wheat, oats, millet, rice, and wheat) used in baby food. Wang et al. [17] established a method for the detection of five *A*Ts in fruits (apple, sweet cherry, tomato, and orange) via a homemade solid-phase extraction (SPE) cartridge step followed by ultrahigh-performance liquid chromatography–tandem mass spectrometry (UPLC–MS/MS); the recovery values were between 74.2% and 102.4%, the relative standard deviation (RSD) was less than 4.7%, and the limits of quantification (LOQ) were in the range of 1–5 μg/kg. However, few analytical *A*T studies have focused on tuberous crops. Only one such study from China was published, where AOH, AME and ALT were tested in 50 samples of sweet potato by using pressure capillary electrochromatography combined with SPE [4]. Tuberous crops under favorable environmental conditions (e.g., high temperature and moisture) during hoarding are prone to germination and molding, and the subsequent contamination by toxins produced by fungi [18]. Once formed, these toxins are stable and not easy to decompose. Food-processing methods and traditional storage criteria have not been able to entirely eliminate toxin pollution in the food delivery chain [19]. Thus, it is essential to monitor the content of *A*Ts using analytical technologies during storage.

To overcome the deficit of research on *A*Ts in tuberous crops and their change during storage, the main challenge is to establish a technology that can perform the rapid and simple extraction of these contaminants from tuberous crops products, tackling issues that arise mainly due to the discrepancy in the physicochemical characters of *A*Ts. Extraction is a key process because it detects the recovery of total toxins in research [20]. UPLC–MS/MS has been an important asset in the analysis of *A*Ts [21]. Triple quadrupole (QqQ) is widely used as a major tool for the qualification and quantification of toxins because of its higher sensitivity, specificity, and effectiveness [22,23]. Among the latest trends that are attractive alternatives, sample pretreatments for detecting trace mycotoxins via UPLC–MS/MS predominantly apply the SPE and quick, easy, cheap, effective, rugged, and safe (QuEChERS) methods [24,25]. Although SPE is valid for eliminating disturbance impurities from matrices, it is time-consuming and complicated. QuEChERS technologies have been successfully applied for the simultaneous determination of toxins in cereals because this rapid and easy process is obviously superior and has high determination throughput [26]. Therefore, the application of QuEChERS methods is essential for monitoring or studying multiple *A*Ts in tuberous crops.

This study aims to establish a reliable and simple UPLC–MS/MS technology for the homochromous assay of AOH, AME, TeA, Ten, ATX-I, ALS, and ALT metabolized by *Alternaria* fungi during storage, coupled with QuEChERS pretreatment for the purification of *A*Ts from tuberous crop samples. The technology was majorized and validated with authentic samples.

## 2. Materials and Methods

### 2.1. Reagents and Chemicals

High-performance liquid-chromatography-grade acetonitrile, methanol, and formic acid (FA) were acquired from Merck Life Science Technology (Nantong) Co., Ltd. (Nantong, China). Analytical-reagent-grade NaCl and anhydrous MgSO_4_ were acquired from Sinopharm Chemical Reagent Co., Ltd., (Shanghai, China). Graphitized carbon black (GCB), primary secondary amine (PSA), and octadecylsilyl (C_18_) were acquired from ANPEL Laboratory Technologies (Shanghai) Inc. (Shanghai, China). Ultrapure water was obtained using a Milli-Q water purification system (Shanghai Motorcycle Science Equipment Co., Ltd., Shanghai, China).

*A*T (Ten, AME, AOH, TeA, ALT, ALS, and ATX-I) standards (purity > 98.0%) were purchased from ANPEL Laboratory Technologies (Shanghai) Inc. (Shanghai, China). First, 100 µg/mL of a standard stock solution of each *A*T was prepared with acetonitrile (refrigerated at –20 °C). Then, 1.0 µg/mL standard solution was prepared by mixing the seven individual standard stock solutions (stored at 4 °C). The mixed standard stock solution was diluted with a blank matrix solution to prepare working standard solutions with final concentrations of 2.5, 5, 10, 25, 50, 100, 250, and 500 μg/L. 

### 2.2. Samples

Tuberous crop samples were obtained from supermarkets in Ningbo, Zhejiang, China. All samples were freshly picked and ensured to be intact without visible rotting parts. In the preliminary treatment, all matrices were directly placed at 4 °C up till germination and the appearance of mold symptoms. Before analysis, the tuber samples were thoroughly homogenized. Second, all the samples were immediately vacuum-packed after lyophilization and stored at 4 °C till analysis.

### 2.3. Toxin Extraction from Tubers Using the QuEChERS Procedure

Samples (3 g) were transferred into a 50 mL plastic centrifuge tube containing 2 g MgSO_4_, 1 g NaCl, and 15 mL of the extractant with 1.5% FA–acetonitrile (*v*/*v*). The mixture was eddied for 1 min, ultrasonically extracted for 10 min, and centrifuged at 9500 rpm and 4 °C for 10 min (step 1). The upper section of the extract was placed in a plastic centrifuge tube containing 150 mg C_18_ adsorbent, eddied for 1 min, and then centrifuged at 4500 rpm for 5 min (step 2). Lastly, the organic-phase extract was dried with nitrogen in a 40 °C water bath (step 3), resolved with 1 mL acetonitrile, and percolated with a polytetrafluoroethylene (PTFE) syringe filter (13 mm, 0.22 μm) (step 4).

### 2.4. Toxin Determination Using UPLC–MS/MS

UPLC–MS/MS (XEVO TQ-XS, Waters Technology (Shanghai) Co., Ltd., Shanghai, China) was used for analysis. Toxins were isolated at 40 °C by using a C18 column (100 × 2.1 mm I.D., 1.7 μm) (Waters Technology (Shanghai) Co., Ltd., Shanghai, China), with a flow rate of 0.4 mL/min. The mobile phase consisted of ultrapure water containing 0.1% FA (Phase A) and acetonitrile (Phase B). The injection volume was 5 μL. A linear gradient was applied: 90:10 to 5:95 for 5 min, 5:95 for 7 min, 90:10 for 7.5 min, and 90:10 for 10 min for mobile Phases A and B (*v*/*v*). 

The mass spectrometer was operated in multiple reaction monitoring (MRM) and full-scan modes for the qualitative and quantitative analyses of the toxins. In MS, the capillary voltage was 1.08 kV, the ion source temperature was 150 °C, desolvation gas temperature was 600 °C, the voltage of radio-frequency (RF) Lenses 1 and 2 was 15.0 V, desolventizing gas flow was 1000 L/h, cone backflushing gas flow was 150 L/h, desolvation gas flow was 900 L/h, and cone gas flow was 150 L/h. Standard solutions of the toxins were estimated in both positive and negative electrospray ionization (ESI) modes.

### 2.5. Evaluation of Recovery and Matrix Effect

Extraction recoveries were assessed by comparing the peak areas obtained from the extracted spiked samples with the extraction recoveries originally spiked in the blank tuberous crop samples with equivalent concentrations. The existence of matrix constituents in the extract (coeluting compounds) affects the ionization of substances when ESI mode is used to produce the MEs [27]. Each compound was detected in a mobile phase (A and B, 50/50, *v*/*v*). The tuber samples were preanalyzed to ensure that they excluded any analytes. Signal inhibition or reinforcement on account of MEs was computed using the following equation.

(1) $$ME\left(\%\right)=\frac{B}{A}\times 100\%$$

where A is the response of the added toxin in pure solvent, and B is the response for the same proportion of the toxin added to the potato matrix.

### 2.6. Method Validation

The validation arguments of the approach, namely, linearity, recovery, intermediate precision (reproducibility), repeatability, LOQ, and limits of detection (LOD), were calculated on the basis of Vogelgesang et al. [28]. Linearity was computed using the coefficient of determination (*R*^2^) of the calibration curves. The LOD and LOQ were detected by contrasting chromatographic signals from the matrices containing low concentrations of the target compounds with those of the blank samples and developing the minimal concentration at which the compound could be reliably determined and quantified, respectively. Signal-to-noise (S/N) ratios of 3:1 and 10:1 were used to calculate the LOD and LOQ, respectively. The minimal, intermediate, and maximal concentrations of the analytical curve for each analyte were applied to obtain the recovery, repeatability, and intermediate precision. Furthermore, repeatability and intermediate precision were computed as the RSD of four analyses determined on six different days (*n* = 24) and six analyses detected on the same day (*n* = 6).

Furthermore, repeatability (RSD_r_) and reproducibility (RSD_wR_) were computed in the laboratory by one and three analysts, respectively.

## 3. Results and Discussion

### 3.1. Optimization of MS/MS Conditions

**Figure 2:**
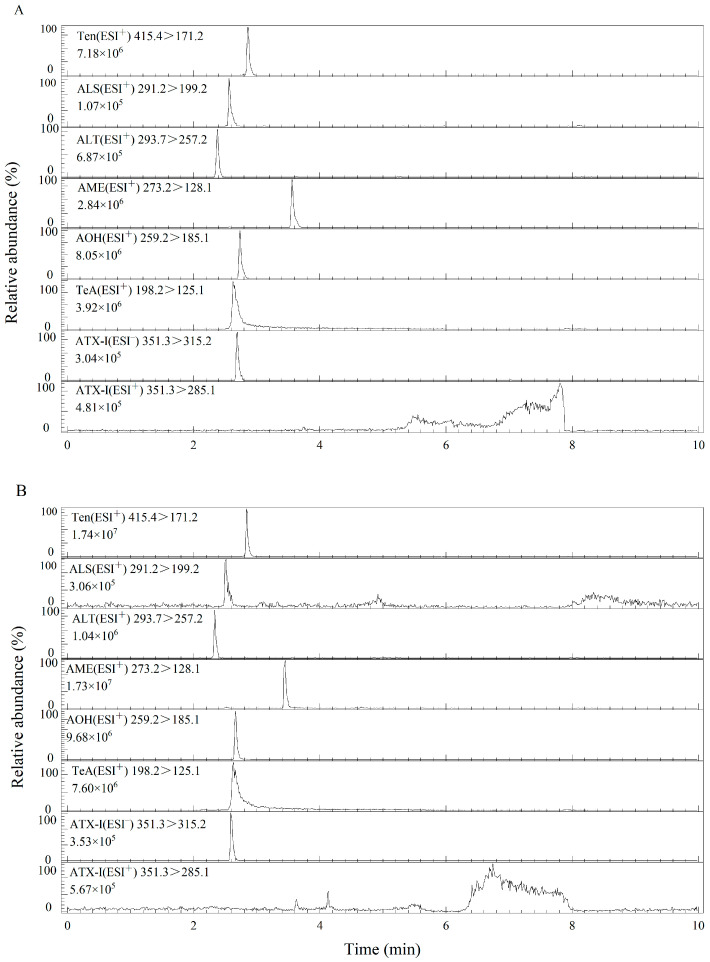
UPLC–MS/MS chromatograms of *Alternaria* toxins standards using (**A**) BEH C18 and (**B**) HSS T3 columns.

Stock solutions (50 μg/L) of the seven *A*T standards were prepared using an acetonitrile/water solution (50/50, *v*/*v*). The ionization mode (ESI^+^/^−^) was determined on the basis of the chemical ionization properties of the seven types of *A*Ts. Six *A*Ts (AOH, AME, TeA, Ten, ALT and ALS) dissolved in the acetonitrile/water solution (50/50, *v*/*v*) could produce [M + H]^+^ parent ions with high abundance in ESI^+^ mode, while ATX-I could generate high responses in ESI^−^ mode (Figure 2). Subsequently, we selected MRM shifts and the optimal response of the relevant acquisition parameters (collision energy and cone voltage) under ESI^+^/^−^ mode conditions by injecting the standard solution into the mobile phase with an injection pump (Table 1).

### 3.2. Optimization of UPLC Conditions

#### 3.2.1. Selection of UPLC Columns

The choice of UPLC columns with quick measurement, high sensitivity, and efficient separation is a precondition for developing multiplex detection methods. Therefore, two LC columns with diverse lengths and particle diameters, that is, (A) the BEH C18 column (100 × 2.1 mm I.D., 1.7 μm, Waters, Milford City, MA, USA) and (B) the HSS T3 column (50 × 2.1 mm I.D., 1.8 μm, Waters, Milford City, MA, USA) were chosen for their isolation efficiency. The isolation efficiency and sensitivity of Column A were much better than those of Column B because column efficiency was obviously enhanced with the increase in surface area per unit volume. 

Furthermore, the hybrid particles used in the BEH C18 column have the advantages of a wide pH range (1–12), lower secondary interactions, faster analysis, higher resolution, better peak shape, and higher column efficiency. Although the HSS T3 column had strong retention capacity, ALT and ALS had no obvious peak shape, and TeA exhibited a tailing phenomenon. Hence, the entire isolation process of the seven *A*Ts was completed in only 10 min by using a BEH C18 column at a 0.4 mL/min flow rate, which fitted well with the MS/MS electrospray ionization conditions (Figure 2).

#### 3.2.2. Mobile Phase Selection

In addition to optimizing the separation efficiency of the chromatographic system for good resolution and high sensitivity, the selection of the mobile phase is crucial, as it affects the ionization efficiency of the compounds before they enter the MS/MS system.

Results of the MS full scan of the seven *A*Ts indicate that AOH, AME, TeA, Ten, ALT, and ALS could generate the corresponding [M + H]^+^ ions with the mobile phase of water/acetonitrile in ESI^+^ electroscopy mode, and ATX-I generated [M + H]^−^ ions under ESI^−^ electroscopic mode. Except for TeA, the responses of the [M + H]^+^ parent ions changed obviously when 0.1% (*v*/*v*) FA or 10 mmol/L ammonium formate was added. Nevertheless, when 10 mmol/L of ammonium formate was added, the abundance and sensitivity of TeA were obviously alleviated in ESI^+^ electroscopic mode. Considering the principles affecting separation efficiency and detection sensitivity, 0.1% (*v*/*v*) FA was a reasonable compromise.

**Figure 3:**
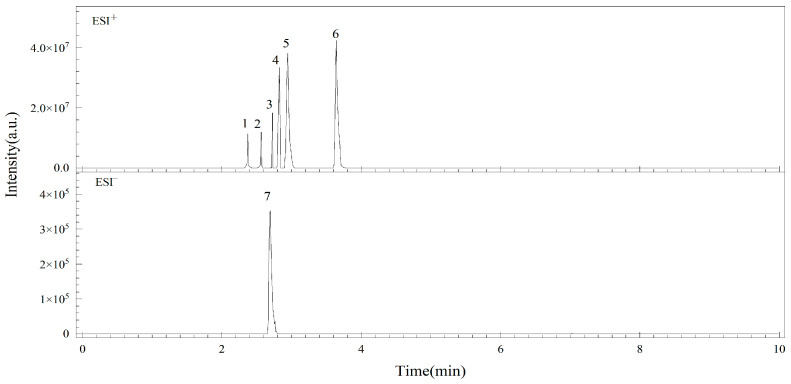
Positive ion chromatogram of six *Alternaria* toxins, and negative ion chromatogram of ATX-I under optimized conditions in a 50 μg/L standard solution. 1: ALT, 2: ALS, 3: TeA, 4: AOH, 5: Ten, 6: AME, 7: ATX-I.

Regarding the option of strongly eluting mobile phase, methanol and acetonitrile were considered as candidates because most mycotoxins are freely soluble in these two solvents [29]. The results show that, when acetonitrile was selected as the mobile phase, the ionization degree of all selected analytes was greatly increased under [M + H]^+^ and [M + H]^−^ electroscopic modes. Thus, acetonitrile was chosen as the strong elution mobile phase for this study. The chromatograms of the seven *A*Ts obtained under optimal conditions show that they could be separated well within 5 min (Figure 3).

### 3.3. Optimization of Sample Pretreatment

**Figure 4:**
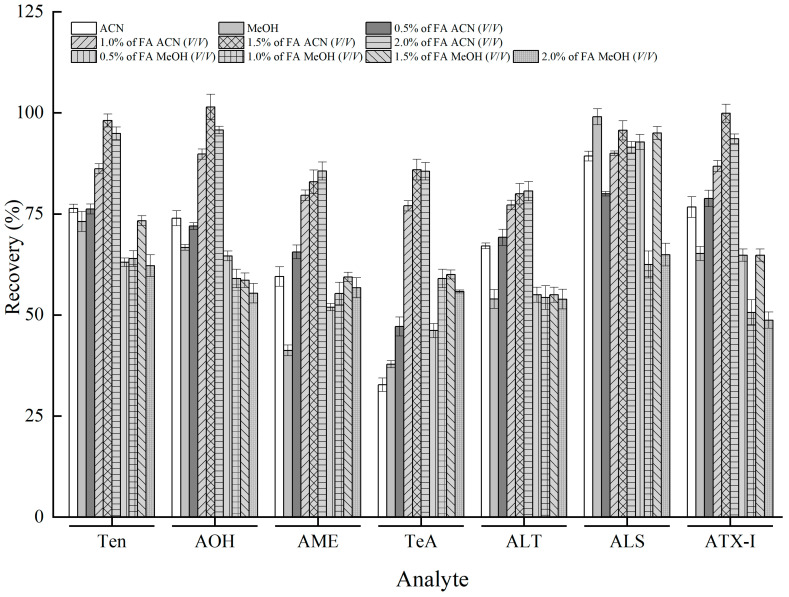
Recoveries of *Alternaria* toxins extracted in different extraction solvents (*n* = 3).

QuEChERS employs a solvent for liquid–liquid partitioning, and uses adsorbents to purify the extracts. This method is popular in food pollutants due to its simplicity, minimal number of steps, and for being able to successfully eliminate interfering compounds from samples [30]. To choose a suitable extraction solvent, methanol and acetonitrile, and different proportions of FA–methanol or FA–acetonitrile solution were successively used on the basis of the solubility of the seven *A*Ts. Moreover, TeA (p*K_a_* = 3.5) is an acidic compound [31], and the pH of the extraction system was lower than p*K_a_* to facilitate the distribution of TeA in the organic phase. After extraction, the samples were dehydrated using MgSO_4_ and stratified via a salting-out procedure using NaCl. The results show that high extraction efficiency and recovery of the selected analytes, which were higher than 70%, were achieved when an FA–acetonitrile solution was used as the extraction solvent (Figure 4). Considering the recommended ratio of FA, a 1.5% FA–acetonitrile solution was selected as the extraction solvent. To assess the optimal extraction solvent, it is invaluable to compare it with other validated reference methods. In such a consideration, we used validated methodologies that mainly included fruits and vegetables extracted with a 1.5% FA–acetonitrile solution, and analyzed with LC–ESI–MS/MS [32] or UPLC–MS/MS [33] to compare them with the current method. In addition, acetonitrile could precipitate proteins, clearing the extract and reducing impurities in the purification step. Therefore, 1.5% FA–acetonitrile was eventually selected as the extraction solvent for pretreatment.

Additionally, extraction recoveries at different ultrasonic and oscillation times (0, 5, 10, 15, and 20 min) were tested. The results indicate that the recoveries of the seven *A*Ts reached the maximal values (80–95%) with an increase in ultrasonic time (10 min) compared to those without ultrasonic or oscillation extraction (0 min), which were only between 35% and 60%. However, the recoveries of the seven *A*Ts were not significantly correlated with oscillation time. Ultrasound has the physical characteristic of promoting the wall breaking or deformation of plant cell tissues, which promotes the transfer of target analytes to the extraction solvent and improves the extraction efficiency [34], which was demonstrated by the addition of ultrasonic extraction for 10 min being beneficial for the extraction of the *A*Ts. Accordingly, the optimal ultrasonic extraction time was 10 min.

### 3.4. Optimization of QuEChERS Clean-Up

Purification is also a critical step that can dramatically influence the qualitative and quantitative performance of the method. The applications of three main adsorbents (PSA, C_18_, and GCB) in QuEChERS has been reported in many studies [33]. Owing to the nature of diversity matrices and analytes, it is vital to choose the correct adsorbents and component ratio. Instead of improving the purification efficiency and elimination of MEs, an unreasonable or redundant adsorbent may result in unsatisfactory recovery.

After extracting the tuberous crop samples in an acetonitrile solution with 1.5% FA, the obtained solution was turbid and exhibited a dark color, probably because impurities, for example, natural pigments, were also extracted into the solution. Therefore, it was essential to further purify the extracts to reduce the effect of the impurities [32]. QuEChERS cleanup techniques have been widely used in the field of the agricultural products and food detection [35], usually employing adsorbents such as C_18_, PSA, and GCB. Bearing that in mind, the purification efficiencies of different adsorbents were compared in this study. 

**Figure 5:**
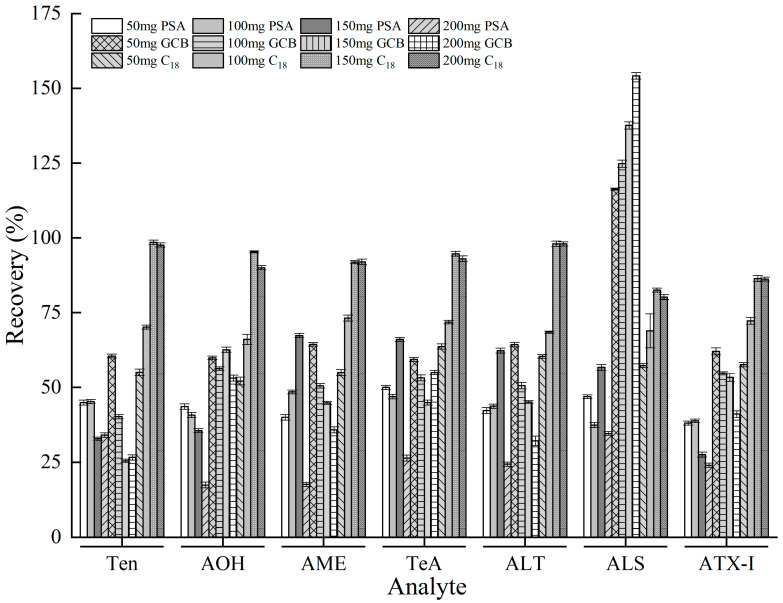
Purification effects with different amounts of adsorbents in QuEChERS clean-up (*n* = 3).

Although pigment removal with the PSA adsorbent was acceptable, the recovery rate was not high. This could be attributed to the violent interaction existing in both PSA and acidic compounds, and the presence of PSA could be maintained with weak anion exchange or polar adsorption (Figure 5) [36]. Moreover, the low recoveries of Ten, AOH, and AME (< 40%) by the GCB adsorbent were similar to those of *A*Ts [37]. The recovery of the target ALS purified with the GCB adsorbent in QuEChERS purification was up to 155.9% (Figure 5), which was mainly related to the matrix reinforcement effect; this is consistent with the results reported by Liu [38].

Compared to the PSA and GCB adsorbents, the adsorbent purification efficiency of C_18_ was significantly better (*p* < 0.05), with satisfactory pigment removal and recovery. Furthermore, 150 mg C_18_ was chosen for purification in this experiment since it achieved the highest purification efficiency (80.7–96.7%).

### 3.5. Method Validation

#### 3.5.1. Calibration and Method Validation

To verify and evaluate this approach, the linear calibration plots of the concentrations of the seven *A*Ts were established. Good linearity and determination coefficient (*R*^2^ > 0.99) were obtained in the concentration range of 2.5–500 μg/L (Table 2). The sensitivity of these target compounds was primarily determined via their ionization efficiency in MRM mode. Noncontaminated tubers were sampled and spiked with the mixed standards of all seven *A*Ts. After sample pretreatment, injection, and detection, the LOD (S/N = 3) and LOQ (S/N = 10) levels were analyzed for the seven *A*Ts with MassLynx v4.0 software. The LOQ of the seven *A*Ts was 0.83–2.31 μg/kg. Similarly, for the intra-/inter- day precision tests, uncontaminated tuberous crops were used with the addition of the mixed standards of the seven *A*Ts.

Repeatability and recovery experiments (Table 2) showed that the average recovery of the samples was in the range of 83.2–104.0% (within a reasonable value of 80–120%) under different spiked concentration levels (low: 1–2.5 μg/kg; medium: 2–5 μg/kg; high: 10–25 μg/kg). The RSDs for the intra- and inter- day tests were 3.52–6.55% and 4.02–7.26%, respectively (within a reasonable limit of less than 10%). Good repeatability was achieved at all spiking levels (*n* = 6). In general, the analytical method had credible repeatability, precision, and accuracy.

#### 3.5.2. Matrix Effect

The method matrix effects (MEs) are where the ionization signal of analytes is suppressed or enhanced in UPLC–MS/MS analysis. Notably, in ESI mode, various physical and chemical processes can affect ionization signals. In particular, MEs are hard to eliminate [33,39].

In the present study, 30 samples (6 samples from each of the following: taro, potato, sweet potato, yam, and cassava) were selected to evaluate the MEs. Each sample was mixed and extracted as previously mentioned (Section 2.5). The reserve fluid was then diluted with pure solvents to prepare a certain concentration of mixed standard work solution to obtain precise results for all matrices.

These solutions were totally determined under optimal instrumentation conditions. MEs were then calculated with Equation (1), which yielded a range of 84.5–133.2% (Table 2). Particularly, the MEs of AME and ATX-I were slightly higher than 120, a fact that can be ignored.

### 3.6. Measurement of Real Samples during Storage

The application of the validated method was to detect and quantify the presence of seven *A*Ts during storage (fresh, germinated, and moldy). We analyzed 30 samples (6 from each of the following: taro, potato, sweet potato, yam, and cassava). The results demonstrate that the detected concentrations of the seven *A*Ts in these samples varied. As listed in Table 3, the main contaminating toxins in the tuberous crops during storage were TeA, AOH, AME, ALS, and Ten. In the absence of germination or mold, the maximal concentrations were 2.13 µg/kg for TeA, 2.74 µg/kg for AOH, 2.01 µg/kg for AME, 9.47 µg/kg for ALS, and 5.08 µg/kg for Ten. After germination, the content of the seven *A*Ts was slightly higher than that at the fresh stage of tubers. The experimental results show that the content of the seven *A*Ts of the tuberous crops was much higher after tubers had molded than that of fresh and germinated tubers after storage (3.87 µg/kg for TeA, 4.73 µg/kg for AOH, 3.25 µg/kg for AME, 14.51 µg/kg for ALS, 7.46 µg/kg for Ten). The frequency of detection in tuberous crops further increased in the rotten tubers, probably due to the progress of *Alternaria* infection increasing the fungal biomass during storage. In summary, ALT was not detected during storage, which was comparative with the results of Yuan et al. [4]. Furthermore, AME often occurs in conjunction with AOH, which is in accordance with the investigation of Yan et al. [40]. ALS was detected in most samples, whereas ATX-I was almost undetectable. TeA was mainly detected in taro and potato, AME and AOH in sweet potato, and Ten in yam tubers.

Notably, the *A*T contamination of tuberous crops takes place during storage. As storage conditions vary, the routine surveillance of the content of *A*Ts in tuberous crops is essential to protect the population from the risk of exposure to these *A*Ts.

### 3.7. Comparison among Analytical Methods for Alternaria Toxins

Table 4 compares the performance of our method with that of similar published methods for the analysis of *A*Ts in tuberous crops and other food products. Both our method and other analytical methods were validated by using spiked recovery studies to obtain recovery and precision data. Furthermore, LOD and/or LOQ are provided. However, our method employed a more convenient, high-throughput QuEChERS method combined with UPLC–MS/MS detection that significantly reduced the sample detection time and improved the detection efficiency compared to SPE and dispersive liquid–liquid microextraction (DLLME). Compared to using the same QuEChERS method, we detected seven *A*Ts simultaneously, which far exceeded those methods that can detect only a few *A*Ts. In addition, our method achieved high recovery percentages (83.2–104.0%). In addition, we plan to develop a simpler and more sensitive UPLC–MS/MS method to detect more *A*Ts in the future.

## 4. Conclusions

A robust and sensitive method for the simultaneous analysis of seven *A*Ts was established with the optimized QuEChERS method coupled with UPLC–MS/MS. The method exhibited excellent analytical performance, satisfactory linearity, high precision, proper accuracy, favorable sensitivity, and negligible MEs. Another advantage was that the method required only 5.0 min of analysis for each analyte and thus enabled the enhanced throughput of samples. This method was successfully applied to monitor the concentrations of the seven *A*Ts during storage (fresh, germinated, and moldy) in tuberous crops (taro, potato, sweet potato, yam, cassava). Specifically, ALT was not detected during storage, and ATX-I was almost undetectable. In contrast, ALS was detected in most samples, while AME often occurred in conjunction with AOH in sweet potato, TeA was mainly detected in taro and potato, and Ten in yam. Their concentrations in fresh, germinated, and moldy tubers were 2.01–14.51 μg/kg and significantly increased during storage.

The determination of the *A*T concentrations in this research could provide a sufficient basis for determining regulatory parameters in tuberous crops. Furthermore, the robust method of *A*T analysis developed in this study provides the means for acquiring reliable data, which is of great significance for supporting the further development of legislation related to these compounds for the protection of the population from health risks that may be associated with these *A*Ts.

## Figures and Tables

**Table 1:** Retention time and MS/MS parameters for the seven *Alternaria* toxins in MRM mode.

Analytes	Retention Time/min	Ionization Mode	Parent Ion (*m*/*z*)	Daughter Ion (*m*/*z*)	Cone Voltage/V	Collision Energy/eV
Ten	2.85	ESI^+^	415.4	132.1 * 115.1	25	13.18
AME	3.55	ESI^+^	259.2	258.2 * 128.1	25	30.25
AOH	2.73	ESI^+^	273.2	185.1 * 213.2	25	40.25
TeA	2.62	ESI^+^	198.2	125.1 * 153.1	25	15.12
ALT	2.37	ESI^+^	293.2	257.2 * 275.4	25	12.8
ALS	2.56	ESI^+^	291.2	227.2 * 255.2	25	30.18
ATX-I	2.68	ESI^−^	353.1	333.3 * 315.25	25	8.10

Note: *, quantitative ions.

**Table 2:** Methodology verification results of the linear relationships, sensitivity, precision, extraction recovery, and matrix effect of *Alternaria* toxins.

Analyte	ME	Linear Range(μg/L)	Linear Equation	*R* ^2^	LOD (μg/kg)	LOQ (μg/kg)	Low Spike Levels			Medium Spike Levels			High Spike Levels		
							Mean Recovery (%) (*n* = 6)	RSD_r_ (%) (*n* = 6)	RSD_wR_ (%) (*n* = 24)	Mean Recovery (%) (*n* = 6)	RSD_r_ (%) (*n* = 6)	RSD_wR_ (%) (*n* = 24)	Mean Recovery (%) (*n* = 6)	RSD_r_ (%) (*n* = 6)	RSD_wR_ (%) (*n* = 24)
TeA	115.02	2.5–500	*y* = 8513.11*x* + 992.03	0.9978	0.27	0.89	96.21	3.56	4.21	97.03	3.67	4.30	102.37	3.77	4.56
AOH	113.90		*y* = 7580.95*x* + 579.78	0.9996	0.25	0.83	95.66	4.21	4.52	94.69	3.96	4.69	97.21	4.06	4.82
AME	133.20		*y* = 16842.20*x* + 3351.28	0.9990	0.70	2.31	92.01	4.35	4.52	93.25	4.69	4.79	95.21	4.58	4.36
ALS	92.71		*y* = 481*x* + 226.58	0.9973	0.32	1.06	93.61	3.52	4.02	94.50	3.69	4.50	96.00	4.01	4.15
ALT	84.50		*y* = 976.24*x* + 102.31	0.9987	0.52	1.72	104.00	5.18	6.31	83.24	5.69	6.29	96.21	5.54	6.58
ATX-I	123.51		*y* = 5015.64*x* + 568.28	0.9997	0.61	2.01	86.65	6.23	6.52	92.51	6.17	6.58	83.20	5.98	6.39
Ten	117.72		*y* = 8277.02*x* + 253.09	0.9939	0.50	1.65	93.21	6.55	7.26	90.51	6.49	7.19	89.03	6.50	3.69

**Table 3:** *Alternaria* toxin contamination and detection frequency of tuberous crop samples during storage (*n* = 6).

Analytes	Taro (μg/kg, %)	Potato (μg/kg, %)	Sweet Potato (μg/kg, %)	Yam (μg/kg, %)	Cassava (μg/kg, %)
	5.14, 16.77 ^1^	-	5.21, 33.33	5.08, 33.33	-
Ten	5.46, 33.33	-	5.38, 33.33	5.54, 33.33	-
	5.61, 33.33	-	6.98, 66.67	7.46, 66.67	-
	-	1.02, 16.77	2.01, 33.33	-	-
AME	-	2.07, 16.77	2.65, 33.33	-	-
	-	2.22, 33.33	3.25, 66.67	-	-
	-	3.01, 16.77	2.74, 33.33	-	2.79, 33.33
AOH	-	3.18, 33.33	2.75, 33.33	-	4.23, 33.33
	-	2.22, 33.33	4.73, 33.33	-	4.01, 66.67
	2.13, 16.77	3.07, 33.33	1.04, 33.33	-	-
TeA	2.21, 16.77	3.33, 33.33	1.61, 33.33	-	-
	2.59, 16.77	3.87, 33.33	2.82, 33.33	-	-
	9.47, 33.33	9.83, 33.33	9.53, 16.77	9.49, 33.33	9.79, 33.33
ALS	5.13, 66.67	10.97, 33.33	9.61, 16.77	9.50, 33.33	9.88, 66.67
	14.51, 66.67	11.01, 33.33	9.81, 33.33	9.57, 83.33	10.55, 83.33
	-	0.05, 16.77	-	-	-
ATX-I	-	0.15, 16.77	-	-	-
	-	0.12, 16.77	-	-	-

^1^ In the order of top to bottom are fresh, germinated, and moldy tubers during storage.

**Table 4:** Performance comparison of analytical methods for *Alternaria* toxins.

Analyte	Sample	Method	Detection Technique	LOD (μg/kg)	LOQ (μg/kg)	Recovery (%)	Ref.
ALT, AOH, AME	Sweet Potato	SPE	Pressure Capillary Electrochromatography	0.10–0.9	0.33–2.97	81.3–103.3	[4]
ALT, AOH, Ten, AME, TeA	Fruits	SPE	UPLC–MS/MS.	0.33	1.09	87.4–100.5	[17]
ALT, AOH, TEN, AME, TeA	Cereals	DLLME	UPLC–MS/MS	0.61–48.20	2.01–120.10	72.7–109.1	[37]
ALT, AOH, Ten, AME	Feed	SPE	UPLC–MS/MS	0.15–3.0	0.50–10.00	97.0–104.8	[41]
AOH, ALT	Barley	QuEChERS	UPLC–MS/MS	0.13–0.30	0.45–1.01	85.7–89.4	[42]
TeA, AME, AOH, ALT, Ten	Edible Vegetable Oil	QuEChERS	UPLC–MS/MS	0.06–0.12	2.00–20.00	80.5–105.2	[43]

## Data Availability

Data sharing is not applicable to this article.
